# Electrophysiological evidences demonstrating differences in brain functions between nonmusicians and musicians

**DOI:** 10.1038/srep13796

**Published:** 2015-09-04

**Authors:** Li Zhang, Weiwei Peng, Jie Chen, Li Hu

**Affiliations:** 1 Key Laboratory of Cognition and Personality (Ministry of Education) and School of Psychology, Southwest University, Chongqing, China; 2 Cognition and Human Behavior Key Laboratory of Hunan Province, Hunan Normal University, Changsha, China

## Abstract

Long-term music training can improve sensorimotor skills, as playing a musical instrument requires the functional integration of information related to multimodal sensory perception and motor execution. This functional integration often leads to functional reorganization of cerebral cortices, including auditory, visual, and motor areas. Moreover, music appreciation can modulate emotions (e.g., stress relief), and long-term music training can enhance a musician’s self-control and self-evaluation ability. Therefore, the neural processing of music can also be related to certain higher brain cognitive functions. However, evidence demonstrating that long-term music training modulates higher brain functions is surprisingly rare. Here, we aimed to comprehensively explore the neural changes induced by long-term music training by assessing the differences of transient and quasi-steady-state auditory-evoked potentials between nonmusicians and musicians. We observed that compared to nonmusicians, musicians have (1) larger high-frequency steady-state responses, which reflect the auditory information processing within the sensory system, and (2) smaller low-frequency vertex potentials, which reflect higher cognitive information processing within the novelty/saliency detection system. Therefore, we speculate that long-term music training facilitates “bottom-up” auditory information processing in the sensory system and enhances “top-down” cognitive inhibition of the novelty/saliency detection system.

Research investigating the differences between nonmusicians and well-trained musicians has a long tradition and has revealed important insights into the possible benefits of long-term music training for musicians, such as enhancement of sensory perception, memory, motor execution, and conscious cognitive processing^1^,^2^,^3^. On the one hand, by using various experimental techniques (e.g., behavioral assessment, electroencephalography (EEG) and magnetic resonance imaging (MRI)) accumulating evidences show that long-term music training can markedly enhance sensorimotor skills. Intuitively, this enhancement is reasonable since playing a musical instrument requires the functional integration of information related to multimodal sensory perception and motor execution^4^,^5^. This functional integration led to increased functional activation of relevant cerebral cortices (e.g., primary auditory cortex) in musicians compared with nonmusicians, when they were instructed to perform tasks requiring the use of motor and auditory skills^6^,^7^. Moreover, structural reorganization induced by long-term music training was consistently observed in auditory, visual, and motor brain areas^4^,^8^,^9^.

On the other hand, due to long-term music training, musicians benefit from generally enhanced cognitive processes^10^, including improved working memory^11^, emotion regulation^12^, error monitoring^13^, and cognitive control^1^. The enhancement of these higher cognitive functions resulting from long-term music training is also reasonable for some practical reasons. First, music appreciation, in itself, can be used to modulate the emotions of listeners^14^. For example, music is commonly used to release stress in daily life^14^,^15^. Second, long-term music training for musicians involves repeated enhancement of attentional focus and cognitive control (e.g., maintain focused attention on music without outside distraction). Thus, musicians often evidence enhanced cognitive control of emotions and attentional focus after long-term music training^1^. “Top-down” cognitive control is likely to be enhanced in musicians who have undergone long-term music training. Note that the enhancement of cognitive control and attentional focus in musicians is publically interesting as these benefits can transfer to various other cognitive domains^1^,^16^. However, evidence demonstrating that long-term music training can modulate higher brain functions is surprisingly rare.

Thus, we aimed to test the hypothesis that long-term music training could not only enhance the auditory information processing, but also modulate the higher cognitive functions (i.e., “top-down” cognitive control). Since it has been widely suggested that auditory-evoked potentials (AEPs) contain neural responses to index both the auditory information processing (e.g., steady-state responses^17^) and the higher cognitive information processing (e.g., low-frequency vertex potentials^18^,^19^,^20^,^21^), this hypothesis was assessed by comparing AEPs, evoked by transient and a modified version of steady-state auditory stimuli (Fig. 1), between nonmusicians and musicians (n = 14 for each group).

## Results

### Differences of neural responses elicited by transient auditory stimuli between nonmusicians and musicians

Figure 2 shows the group-level average transient AEP waveforms (FCz-A1A2) and the scalp topographies at the peak latencies of N1 and P2 for both nonmusicians and musicians (n = 14 for each group). Scalp topographies of both N1 and P2 were remarkably similar between nonmusicians and musicians. The N1 was maximal at fronto-central region and extended bilaterally towards fronto-temporal regions, and the P2 was more centrally distributed at the fronto-central region^22^. Whereas both N1 latencies and amplitudes were not significantly different between nonmusicians and musicians (N1 latency: 105 ± 13 ms vs. 115 ± 19 ms, P = 0.13; N1 amplitude: −6.65 ± 2.43 μV vs. −5.37 ± 2.02 μV, P = 0.14), both P2 latencies and amplitudes were significantly different between the two groups (P2 latency: 174 ± 16 ms vs. 200 ± 29 ms, P = 0.008; P2 amplitude: 5.91 ± 3.47 μV vs. 3.29 ± 1.63 μV, P = 0.01). Similar results were obtained when the N1 and P2 amplitudes (i.e., mean peak amplitudes) were measured by calculating the mean values within their respective peak intervals (N1: 80–120 ms; P2: 155–180 ms). Whereas mean peak N1 amplitudes were not significantly different between nonmusicians and musicians (−4.23 ± 2.00 μV vs. −3.04 ± 2.33 μV; P = 0.16), mean peak P2 amplitudes were significantly different between the two groups (4.65 ± 2.89 μV vs. 1.46 ± 2.57 μV; P = 0.005).

To demonstrate that the selection of a single electrode (i.e., FCz) for the statistical analysis was reasonable (i.e., the findings obtained from exploring the brain responses at other electrodes, e.g., temporal electrodes, could be mostly detected at FCz), we performed the same statistical comparisons of transient AEPs measured at bilateral temporal electrodes (i.e., T7 and T8). Peak amplitudes of N1 and P2 waves were not significantly different between nonmusicians and musicians at both electrodes (N1 measured at T7: −3.5 ± 1.5 μV vs. −2.8 ± 0.9 μV; P = 0.13; P2 measured at T7: 2.2 ± 1.7 μV vs. 1.3 ± 0.7 μV; P = 0.08; N1 measured at T8: −3.1 ± 1.7 μV vs. −2.9 ± 0.9 μV; P = 0.8; P2 measured at T8: 2.1 ± 1.6 μV vs. 1.5 ± 1.0 μV; P = 0.2). In contrast, peak latencies of N1 waves were significantly different between nonmusicians and musicians at T8 (116 ± 13 ms vs. 129 ± 18 ms; P = 0.04), but not at T7 (112 ± 11 ms vs. 123 ± 22 ms; P = 0.09). Peak latencies of P2 waves were significantly different between nonmusicians and musicians at T7 (184 ± 15 ms vs. 202 ± 21 ms; P = 0.02), but not at T8 (188 ± 18 ms vs. 202 ± 19 ms; P = 0.054).

The top panel of Fig. 3 shows the group-level average baseline-corrected time-frequency distributions (TFDs) obtained from single-trial AEPs (auditory-induced responses) and single-subject average AEPs (auditory-evoked responses), as well as group-level average PLVs (FCz-A1A2; from top to bottom) for nonmusicians and musicians. Being elicited by transient auditory stimuli, all TFDs contained clear responses located at 0–300 ms and 1–20 Hz, as well as at 0–100 ms and 30–100 Hz. These two time-frequency responses, which were phase-locked to auditory stimuli (showed in phase locking values, PLVs), corresponded to late-latency and early/middle-latency AEPs respectively in the time domain (Fig. 2). Region of interest (ROI) based statistical analysis revealed that the time-frequency regions showed significant differences of both evoked TFDs (ROI1) and PLVs (ROI2) between post-stimulus responses and pre-stimulus responses as well as between nonmusicians and musicians at around 0–300 ms and 1–20 Hz (i.e., late-latency AEPs; Fig. 3, top panel; marked in purple). There were similar scalp topographies of evoked TFDs between nonmusicians and musicians within ROI1 (maximal at fronto-central region, Fig. 3, upper part of the bottom panel); however, permutation testing (5000 times) indicated that the measured magnitudes were significantly larger for nonmusicians than musicians (0.35 ± 0.22 μV^2^ vs. 0.14 ± 0.07 μV^2^; P = 0.002). Within ROI2 (Fig. 3, lower part of the bottom panel), permutation testing indicated that the measured PLVs were significantly larger for nonmusicians than for musicians (0.33 ± 0.08 vs. 0.22 ± 0.05; P < 0.001).

### Differences of neural responses elicited by descending trains of quasi-steady-state auditory stimuli between nonmusicians and musicians

**Figure 1:**
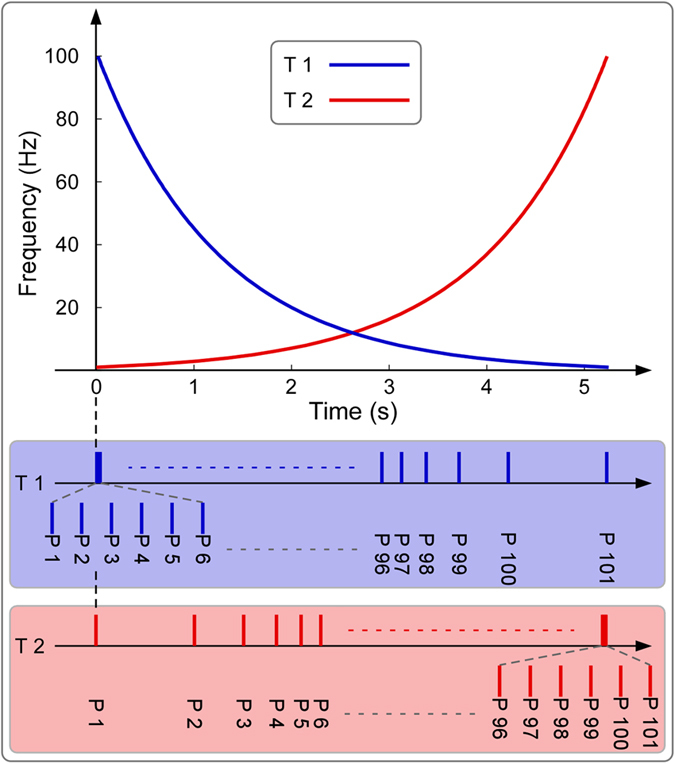
Quasi-steady-state auditory stimuli. The quasi-steady-state auditory stimuli, presented at a comfortable listening level (~80 dB SPL) through binaural earphones, consisted of trains of 1 ms monotone pulses (101 pulses for each train, i.e., P1, P2, …, P101). Two types of train, i.e., descending train and ascending train, are respectively marked in blue and red. In the descending train, the inter-pulse intervals (IPIs), which were changed from 10 ms to 1000 ms, were 1000/100 ms between P1 and P2, 1000/99 ms between P2 and P3, 1000/98 ms between P3 and P4, …, 1000/1 ms between P100 and P101. In this case, the stimulus frequencies were 100, 99, 98, …, 1 Hz for the consecutive pulses. In the ascending train, the IPIs, which were changed from 1000 ms to 10 ms, were 1000/1 ms between P1 and P2, 1000/2 ms between P2 and P3, 1000/3 ms between P3 and P4, …, 1000/100 ms between P100 and P101. The stimulus frequencies were 1, 2, 3, …, 100 Hz for the consecutive pulses in this type of train.

Figure 4 shows the group-level average AEP waveforms (elicited by descending trains of quasi-steady-state auditory stimuli), baseline-corrected TFDs obtained from single-trial AEPs (auditory-induced responses) and single-subject average AEPs (auditory-evoked responses), as well as group-level average PLVs (FCz-A1A2; from top to bottom) for nonmusicians and musicians. All TFDs comprised clear responses located at low frequencies (i.e., 1–20 Hz) and high frequencies (i.e., 30–100 Hz). The low-frequency responses were phase-locked to each pulse of the quasi-steady-state auditory stimuli (showed in PLVs; Fig. 4, fourth row) and corresponded to late-latency AEPs in the time domain (Fig. 4, first row). Even the high-frequency responses were also phase-locked to each pulse of the quasi-steady-state auditory stimuli; these high frequency responses were composed of not only the transient responses (early/middle-latency AEPs in the time domain; Fig. 4, first row), but also the quasi-steady-state responses, which strictly followed the frequency profile of the stimuli (Fig. 1, blue curve). ROI-based statistical analysis revealed that the time-frequency region showing a significant difference of evoked TFDs (ROI1) between post-stimulus responses and pre-stimulus responses as well as between nonmusicians and musicians was observed at 4356–4478 ms and 1–11 Hz (Fig. 4, third row; marked in purple). Within ROI1 (Fig. 5, top panel), permutation testing indicated that the measured magnitudes were significantly larger for nonmusicians than musicians (0.12 ± 0.10 μV^2^ vs. 0.05 ± 0.03 μV^2^; P = 0.014). ROI-based statistical analysis also revealed that the time-frequency regions showing a significant difference of PLVs between post-stimulus responses and pre-stimulus responses as well as between nonmusicians and musicians was observed at 632–1016 ms and 42–62 Hz (ROI2); 4318–4492 ms and 1–12 Hz (ROI3); 28–152 ms and 36–50 Hz, 2432–2584 ms and 30–50 Hz, 3810–3918 ms and 1–13 Hz, 4136–4334 ms and 40–50 Hz, 5294–5412 ms and 1–22 Hz (other ROIs; Fig. 4, fourth row; marked in purple). Since strikingly similar results were observed among low frequency ROIs, and among high frequency ROIs, only the ROI with the largest number of significant time-frequency pixels in the low-frequency region (<30 Hz; i.e., ROI3) and the ROI with the largest number of significant time-frequency pixels in the high-frequency region (≥30 Hz; i.e., ROI2) were illustrated. Within ROI2 (Fig. 5, middle panel), permutation testing indicated that the measured PLVs were significantly smaller for nonmusicians than musicians (0.31 ± 0.12 vs. 0.44 ± 0.11; P = 0.009). Within ROI3 (Fig. 5, bottom panel), permutation testing indicated that the measured PLVs were significantly larger for nonmusicians than musicians 0.22 ± 0.07 vs. 0.14 ± 0.05; P = 0.001).

### Differences of neural responses elicited by ascending trains of quasi-steady-state auditory stimuli between nonmusicians and musicians

Figure 6 shows the group-level average AEP waveforms (elicited by ascending trains of quasi-steady-state auditory stimuli), baseline-corrected TFDs obtained from single-trial AEPs (auditory-induced responses) and single-subject average AEPs (auditory-evoked responses), as well as group-level average PLVs (FCz-A1A2; from top to bottom) for nonmusicians and musicians. All TFDs comprised clear responses located at low frequencies (i.e., 1–20 Hz) and high frequencies (i.e., 30–100 Hz). The low-frequency responses were phase-locked to each pulse of the quasi-steady-state auditory stimuli (showed in PLVs; Fig. 6, fourth row) and corresponded to late-latency AEPs in the time domain (Fig. 6, first row). Even the high-frequency responses were phase-locked to each pulse of the quasi-steady-state auditory stimuli; the high frequency responses were made up for both the transient responses (early/middle-latency AEPs in the time domain; Fig. 6, first row) and the quasi-steady-state responses, which strictly followed the frequency profile of the stimuli (Fig. 1, red curve). ROI-based statistical analysis revealed that the time-frequency region showing a significant difference of evoked TFDs (ROI1) between post-stimulus responses and pre-stimulus responses as well as between nonmusicians and musicians was observed at 1626–1716 ms and 1–17 Hz (Fig. 6, third row; marked in purple). Within ROI1 (Fig. 7, top panel), permutation testing indicated that the measured magnitudes were significantly larger for nonmusicians than musicians 0.15 ± 0.10 μV^2^ vs. 0.06 ± 0.04 μV^2^; P = 0.001). ROI-based statistical analysis also revealed that the time-frequency regions showing a significant difference of PLVs between post-stimulus responses and pre-stimulus responses as well as between nonmusicians and musicians was observed at 4456–4828 ms and 39–59 Hz (ROI2); 992–1326 ms and 1–14 Hz (ROI3); 1602–1748 ms and 1–18 Hz, 1932–2096 ms and 1–15 Hz, 2360–2534 ms and 1–11 Hz (other ROIs; Fig. 6, fourth row; marked in purple). Since strikingly similar results were observed among low frequency ROIs, only the ROI with the largest number of significant time-frequency pixels in the low-frequency region (<30 Hz; i.e., ROI3) was selected for demonstration. Within ROI2 (Fig. 7, middle panel), permutation testing indicated that the measured PLVs were significantly smaller for nonmusicians than musicians 0.28 ± 0.12 vs. 0.40 ± 0.11; P = 0.007). Within ROI3 (Fig. 7, bottom panel), permutation testing indicated that the measured PLVs were significantly larger for nonmusicians than musicians 0.28 ± 0.07 vs. 0.19 ± 0.04; P = 0.002).

## Discussion

Using a quasi-steady-state experimental paradigm, musicians showed significantly larger PLVs of steady-state AEPs at high frequencies (40–60 Hz; Figs 4 and 6) than nonmusicians, which confirmed the notion that long-term music training can enhance the auditory information processing in the sensory system^7^,^23^. In contrast, being evoked by both transient and quasi-steady-state auditory stimuli, musicians showed significantly lower magnitudes and PLVs of AEPs at low frequencies (1–20 Hz; Figs 3,4 and 6) than nonmusicians, which may indicate that long-term music training can enhance the “top-down” cognitive inhibition to the novelty/saliency detection system^24^. In other words, long-term exposure to music and music training most likely increases musicians’ ability to effectively process the sensory information evoked by external auditory stimuli, and to initiate “top-down” cognitive control.

### The enhancement of “bottom-up” auditory processing in musicians

Since the playing of music instruments requires the multimodal integration of sensory, motor, and cognitive information processing in the human brain, continual practice and repetition of such skills over a long period of time should contribute to cortical reorganizations in multiple brain regions^25^, including auditory cortex^7^,^23^, visual cortex^4^, motor cortex^6^, and cerebellum^9^. In support of this postulate, functional enhancement was observed in violonists in the primary somatosensory cortex in resopnse to tactile stimulation^6^, the primary auditory cortex (assessed by N19m-P30m middle-latency AEPs and 40-Hz steady-state AEPs)^23^,^26^ and the auditory associated areas^26^,^27^. These differences between musicians and nonmusicians are likely to be caused by the adaptation/neuroplasticity of long-term music training, which modifies synaptic connections or neural growth processes^9^,^27^,^28^.

**Figure 2:**
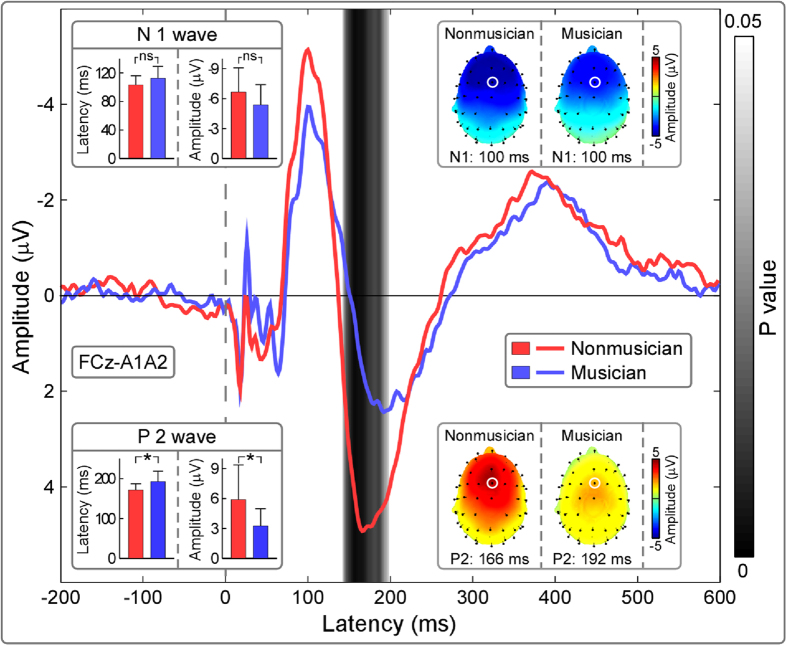
The comparison of event-related potentials (ERPs) evoked by transient auditory stimuli between nonmusicians and musicians. ERPs evoked by transient auditory stimuli (group-level average; FCz-A1A2) from nonmusicians and musicians are respectively marked in red and blue. x axis, latency (ms); y axis, amplitude (μV). The scalp topographies of N1 and P2 in auditory ERPs, from both nonmusicians and musicians, are displayed in the upper and lower parts respectively. Gray scale represents the P values obtained for each time point using an independent sample t-test to assess the significant difference of auditory ERPs between nonmusicians and musicians. Whereas N1 latencies and amplitudes were not significantly different between nonmusicians and musicians, P2 latencies were significantly shorter and P2 amplitudes were significantly larger for nonmusicians than musicians.

**Figure 3:**
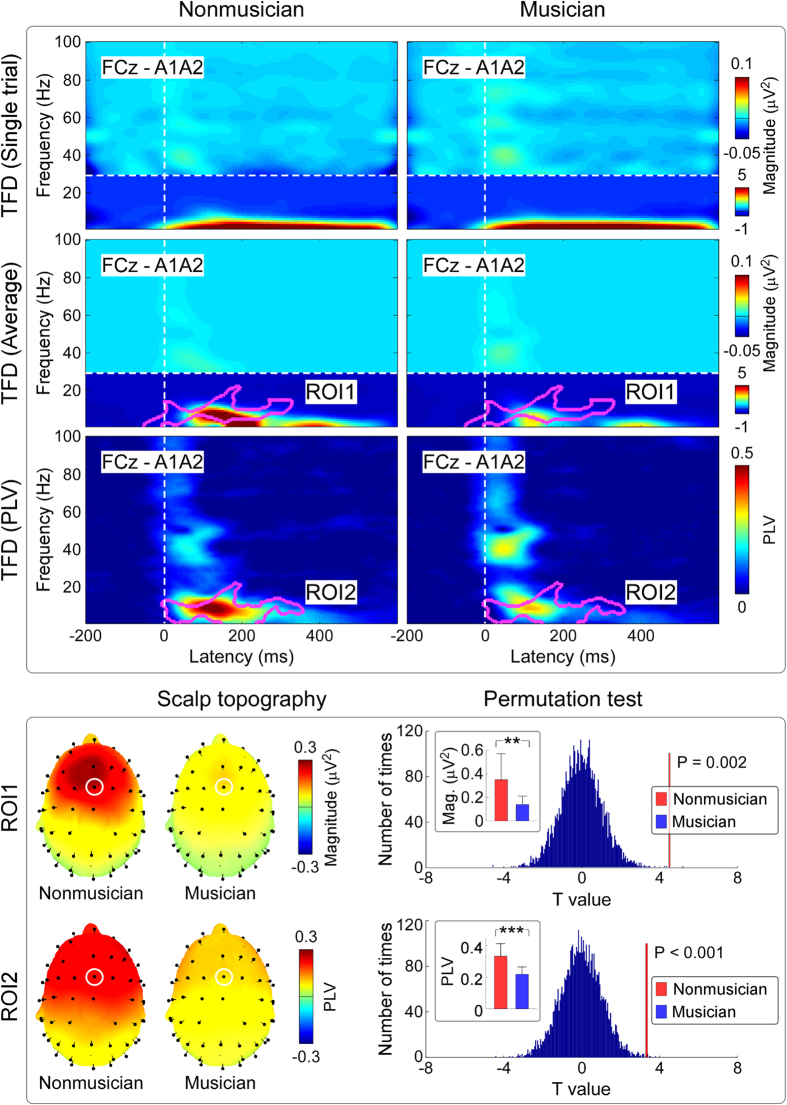
The comparison of time-frequency distributions (TFDs) elicited by transient auditory stimuli between nonmusicians and musicians. *Top panel*: Being elicited by transient auditory stimuli, TFDs of auditory-induced responses (single trial), auditory-evoked responses (average), and phase-locking values (PLVs) (group-level average; FCz-A1A2) are displayed from top to bottom for nonmusicians (left) and musicians (right) respectively. x axis, latency (ms); y axis, frequency (Hz). The region-of-interests (ROIs), outlined in purple curves, had (1) significantly different TFD values than those within the pre-stimulus interval and (2) significantly different TFD values between nonmusicians and musicians. *Bottom left panel*: The scalp topographies, measured from the corresponding ROIs of evoked TFDs (ROI1) and PLVs (ROI2), are respectively displayed in the upper and lower parts for nonmusicians (left) and musicians (right). *Bottom right panel*: Statistical t values and corresponding null distributions within the ROIs of evoked TFDs (ROI1) and PLVs (ROI2) are displayed in the upper and lower parts respectively. Null distributions were generated from 5000 random permutations from all datasets. Statistical t values are indicated by vertical red lines. Within ROI1, permutation tests showed that the t value of the comparison of evoked TFDs between nonmusicians and musicians was significantly different from chance level (P = 0.002). Within ROI2, permutation tests showed that the t value of the PLV comparison between nonmusicians and musicians was significantly different from chance level (P < 0.001).

**Figure 4:**
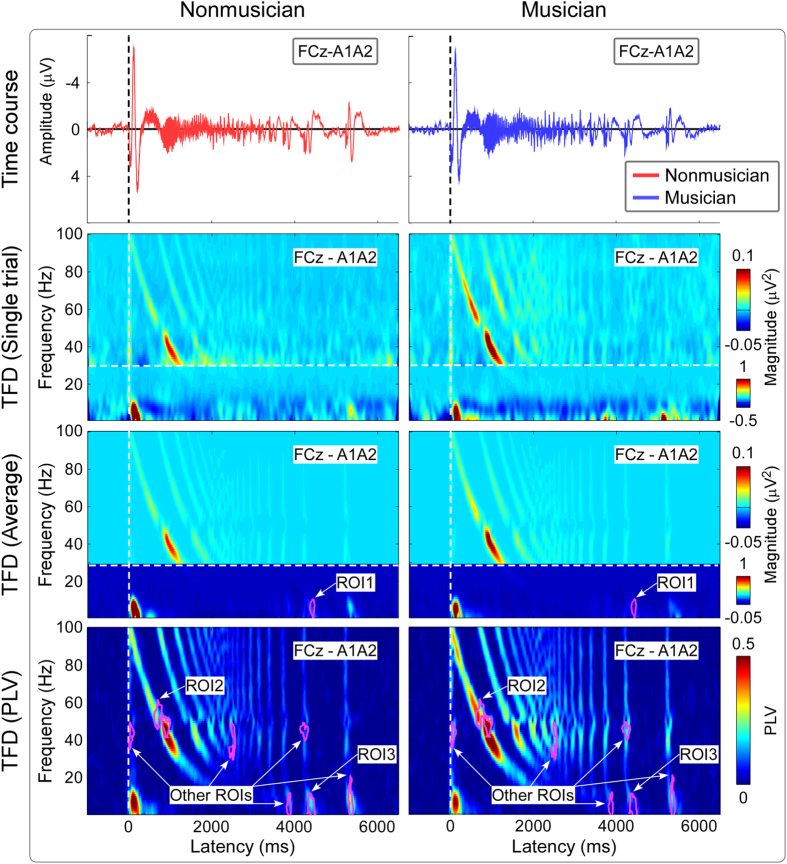
The comparison of neural responses elicited by descending trains of quasi-steady-state auditory stimuli between nonmusicians and musicians. ERPs and TFDs of auditory-induced responses (single trial), auditory-evoked responses (average), and PLVs (group-level average; FCz-A1A2) are displayed from top to bottom for nonmusicians (left) and musicians (right) respectively. The region-of-interests (ROIs), outlined in purple curves, had (1) significantly different TFD values than those within the pre-stimulus interval and (2) significantly different TFD values between nonmusicians and musicians.

In our study, we did not observe a significant difference of early/middle-latency AEPs (and high-frequency responses) between nonmusicians and musicians; in fact, musicians displayed a trend of response enhancement (Figs 2 and 3). Instead, using a quasi-steady-state experimental paradigm, we observed significantly larger PLVs of steady-state AEPs at 632–1016 ms and 42–62 Hz (ROI2 in Fig. 4), and at 4456–4828 ms and 39–59 Hz (ROI2 in Fig. 6) in musicians than nonmusicians. This finding is similar with a previous study^26^, in which the phase of 40-Hz steady-state AEPs was modified by music training, thus indicating that the temporal properties of the neural representations of steady-state AEPs (Heschl’s gyrus in the primary auditory cortex) were affected by training^26^. Considering that PLVs used in the current study measured the phase synchrony of brain responses among different trials^29^, the enhancement of PLVs of steady-state AEPs in musicians, as compared to nonmusicians, indicated that the neural populations in the auditory system responded more synchronously to the onset of auditory stimuli appearing at different times. Also considering that the “bottom-up” auditory processing involves the processsing of incoming auditory stimuli and feature extraction of acoustic signals^30^, the long-term music training could enhance the “bottom-up” auditory information processing in the sensory system by coding the temporal features of the auditory stimuli more synchronously.

40-Hz steady-state AEPs and N19m-P30m middle-latency AEPs are commonly observed at Heschl’s gyrus and may reflect similar neural processing^27^,^31^. In contrast, significant enhancement of PLVs due to music training was only observed from steady-state AEPs, but not from early/middle-latency responses of transient AEPs. This observation could be due to the higher signal-to-noise ratio of steady-state responses than transient responses^17^,^32^, and the large individual variability of the brain responses in both groups (e.g., the existence of some outliers). Note that the modified version of steady-state experimental paradigm proposed in the present study made it possible to explore the steady-state AEPs in a wide range of frequencies (1–100 Hz) without any prior assumptions (Fig. 1). The validity of the proposed quasi-steady-state paradigm was also confirmed by the following two observations. First, the TFDs (both magnitudes and PLVs) of quasi-steady-state AEPs strictly followed the frequency profiles of the auditory stimuli (Figs 4 and 6). Second, strikingly similar results (significantly larger PLVs of steady-state AEPs in musicians than nonmusicians were observed at similar frequenices, i.e., around 40–60 Hz for both types of responses) were obtained from quasi-steady-state AEPs that were elicited by descending and ascending trains of quasi-steady-state stimuli. Indeed, the proposed quasi-steady-state experimental paradigm is not the only way to assess the effect of music training in a wide range of frequencies. Future studies should consider analyzing variations/modifications of the quasi-steady-state experimental paradigm (e.g., changing the frequency range to be explored and changing the frequency profile), which may capture certain distinct advantages.

### The enhancement of “top-down” cognitive inhibition in musicians

**Figure 5:**
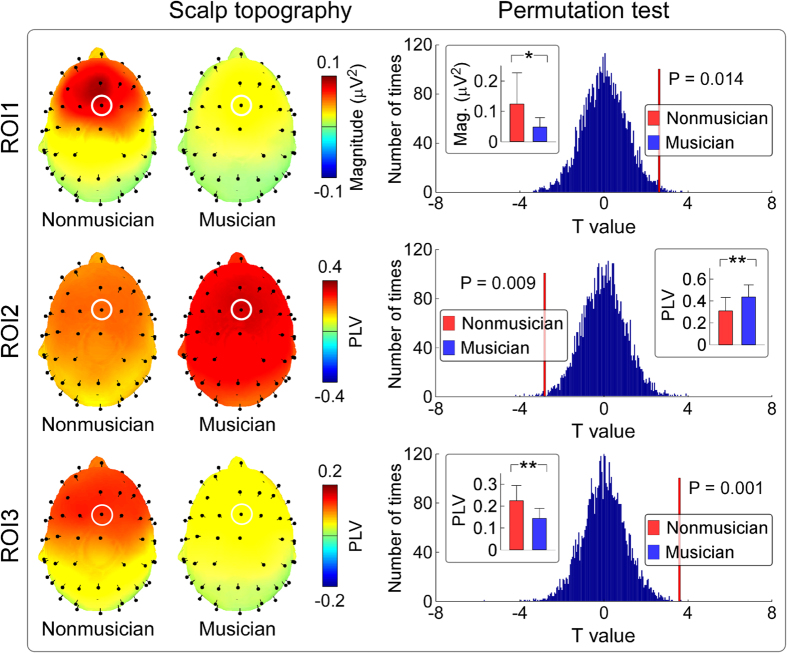
ROI analysis comparing neural responses elicited by descending trains of quasi-steady-state auditory stimuli between nonmusicians and musicians. *Left panel*: The scalp topographies, measured from the corresponding ROIs of evoked TFDs (ROI1) and PLVs (ROI2 and ROI3; outlined in Fig. 4), are displayed from top to bottom for nonmusicians (left) and musicians (right) respectively. *Right panel*: Statistical t values and corresponding null distributions within the ROIs of evoked TFDs (ROI1) and PLVs (ROI2 and ROI3) are displayed from top to bottom. Null distributions were generated from 5000 random permutations from all datasets. Statistical t values are indicated by vertical red lines. Within ROI1, permutation tests showed that the t value of the comparison of evoked TFDs between nonmusicians and musicians was significantly different from chance level (P = 0.014). Within ROI2 and ROI3, permutation tests showed that the t values of the PLV comparisons between nonmusicians and musicians were significantly different from chance level (P = 0.009 and P = 0.001 respectively).

**Figure 6:**
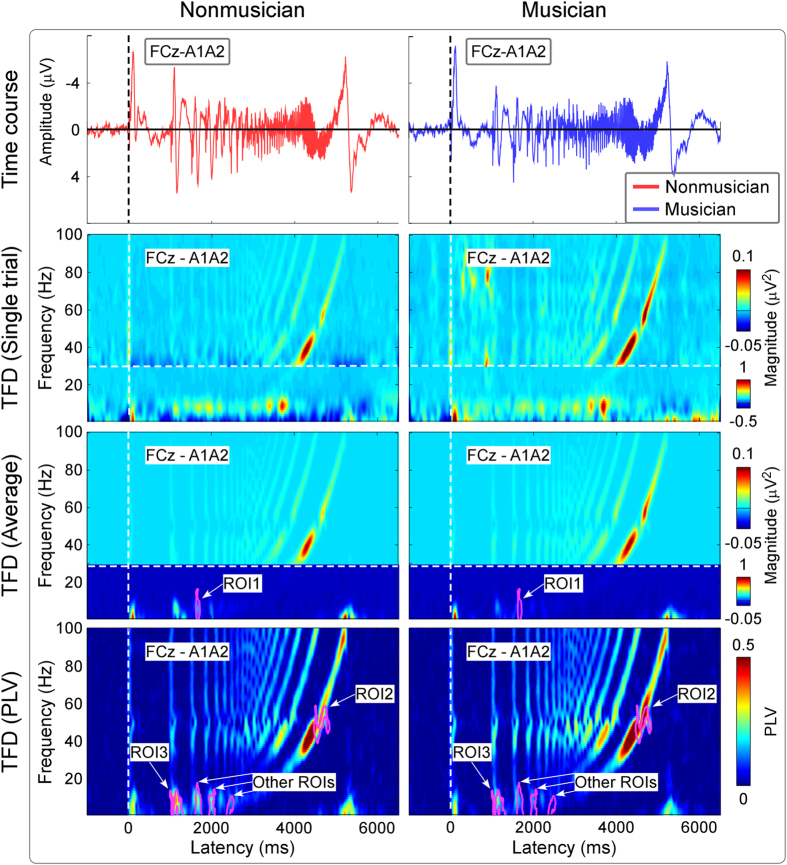
The comparison of neural responses elicited by ascending trains of quasi-steady-state auditory stimuli between nonmusicians and musicians. ERPs and TFDs of auditory-induced responses (single trial), auditory-evoked responses (average), and PLVs (group-level average; FCz-A1A2) are displayed from top to bottom for nonmusicians (left) and musicians (right) respectively. The region-of-interests (ROIs), outlined in purple curves, had (1) significantly different TFD values than those within the pre-stimulus interval and (2) significantly different TFD values between nonmusicians and musicians.

**Figure 7:**
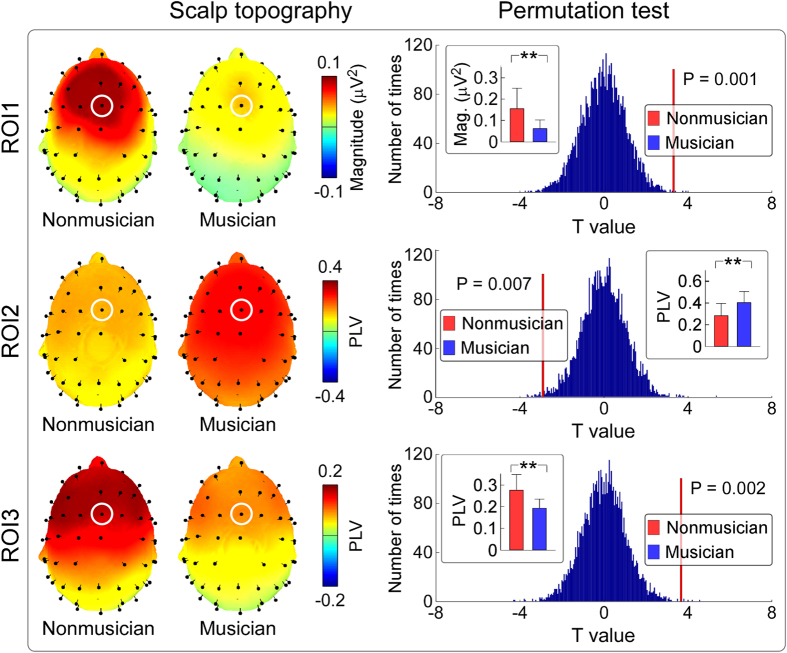
ROI analysis comparing neural responses elicited by ascending trains of quasi-steady-state auditory stimuli between nonmusicians and musicians. *Left panel*: The scalp topographies, measured from the corresponding ROIs of evoked TFDs (ROI1) and PLVs (ROI2 and ROI3; outlined in Fig. 6), are displayed from top to bottom for nonmusicians (left) and musicians (right) respectively. *Right panel*: Statistical t values and corresponding null distributions within the ROIs of evoked TFDs (ROI1) and PLVs (ROI2 and ROI3) are displayed from top to bottom. Null distributions were generated from 5000 random permutations from all datasets. Statistical t values are indicated by vertical red lines. Within ROI1, permutation tests showed that the t value of the comparison of evoked TFDs between nonmusicians and musicians was significantly different from chance level (P = 0.001). Within ROI2 and ROI3, permutation tests showed that the t values of the PLV comparisons between nonmusicians and musicians were significantly different from chance level (P = 0.007 and P = 0.002 respectively).

In the time domain, transient auditory stmuli evoked significantly shorter P2 latency and larger P2 amplitude in nonmusicians than in musicians (Fig. 2). In the time-frequency domain, transient and steady-state auditory stimuli evoked significantly greater magnitudes and PLVs in the low frequencies (1–20 Hz) in nonmusicians than in musicians (Figs 3, 4, 5, 6, 7). Since the low-frequency response was time- and phase-locked to the onset of auditory stimuli, this response corresponded to the brain responses that were detected in the time domain using standard across-trial averaging (i.e., vertex potentials, especially P2 in late-latency AEPs)^33^. As a result, the above findings indicate that long-term music training can suppress auditory-evoked multimodal vertex potentials (especially P2, which was primarily generated from anterior cingulate cortex, ACC)^22^. The vertex potentials (negative-positive biphasic wave, N1-P2 in AEPs) can be elicited by stimuli of various sensory modalities (e.g., auditory, visual, somatosensory)^34^,^35^; regardless of the sensory modality of the applied stimuli, the vertex potentials capture remarkedly similar shape, scalp topography, and sensitivity to the experimental factors^22^. For this reason and also considering that the magnitudes of vertex potentials correlated with the subjective rating of saliency, the vertex potentials are suggested to involve bottom-up cognitive mechamisms of saliency-detection, arousal, or attentional reorientation^22^,^24^,^36^,^37^.

Indeed, this bottom-up hypothesis explained perfectly the variability of vertex potentials at the within-subject level. For example, delievering identical stimuli repeatedly at a constant inter-stimulus interval (i.e., increase the temporal expectancy of the stimulus but decrease the saliency of the stimulus) can significantly reduce the magnitude of vertex potentials for each subject^38^. However, this bottom-up hypothesis cannot explain the variability of vertex potentials at the cross-subject level, as different subjects tend to evidence different vertex potential magnitudes in response to identical stimuli and experimental settings. In addition, the low-frequency vertex potentials, especially the P2 wave, were highly sensitive to some higher cognitive functions (e.g., emotion and affection)^18^,^19^,^20^,^21^. In our study, we observed that the vertex potentials evoked by the same auditory stimuli were significantly smaller in musicians than nonmusicians (Figs 3, 4, 5, 6, 7). This difference between nonmusicians and musicians cannot be explained by bottom-up cognitive mechamisms, since (1) the same auditory stimuli were delieved to both groups in the same experimental settings with the same instructions, and (2) enhanced “bottom-up” auditory information processing in the sensory system was observed in musicians compared with nonmusicians (expressed by PLVs of steady-state AEPs from 40 to 60 Hz).

Instead, the significant difference of vertex potentials between nonmusicians and musicians can be explained by certain “top-down” factors, considering that (1) the neuroplasticity to long-term music training was not only observed in the sensory systems^7^,^23^ but also in the cognitive control systems^1^,^39^, and (2) “top-down” processing is based on prior knowledge of the significance of sensory inputs^30^. This top-down control hypothesis is repeatedly linked to functional variation of the prefrontal cortex after long-term music training^1^,^5^. The cognitive control ability, in general, was enhanced in musicians, which contributed to their improved ability in various aspects, e.g., verbal memory and nonverbal reasoning^16^,^39^. Different from previous studies in which subjects listened passively to tonal stimuli^1^,^27^, subjects in the present study were instructed to focus their attention on auditory stimuli of 1-ms monotone pulses, which sounded like the noise generated by a motorcycle. The observation that the amplitude of multimodal vertex potentials (especially P2) were significantly lower in musicians than nonmusicians (Figs 3, 4, 5, 6, 7) could thus be explained by the enhancement of top-down cognitive inhibition for two reasons. First, the noise-like auditory stimuli could induce the negative emotions. Second, it has been documented that demanding musical training reinforces musicians’ cognitive control abilities^1^. The enhanced cognitive control (i.e., top-down cognitive inhibition) of musicians could help suppress the stimulus-evoked negative emotions, thus suppressing the low-frequency vertex potentials (especially P2 wave). However, more evidence should be provided to further verify this top-down cognitive inhibition hypothesis in the future.

To sum up, we observed that long-term music training enhances the PLVs of steady-state AEPs at high frequencies, but suppresses the magnitudes and PLVs of transient AEPs at low frequencies. These findings can be explained by long-term music training induced neuroplasticity, which contributed to the enhancements of “bottom-up” auditory processing within the sensory system and “top-down” cognitive inhibition to the novelty/saliency detection system. The relationship between the “bottom-up” auditory processing and the “top-down” cognitive inhibition is not clear; our study demonstrated that extending beyond the facilitation of auditory information processing^40^, the long-term music training may also enhance individual cognitive functions^1^. These findings thus suggest that, especially for adolescence and young adulthood, long-term music training may bring important biological benefits.

## Methods

### Subjects

Fourteen nonmusicians (aged 21.0 ± 1.04 years; 6 females) and fourteen musicians (aged 20.4 ± 2.14 years; 5 females) participated in the study. All subjects, who were undergraduate and graduate students from Southwest University (Chongqing, China), were healthy, right-handed volunteers with normal hearing. Nonmusicians and musicians did not differ on sex distribution, age, hearing, and educational attainment (Table 1). Musicians, who reported formal music training with different instruments (none of the musicians were trained with percussion instruments), started training at the age of 10.4 ± 3.72, for 9.07 ± 4.68 years (Table 1). All subjects gave written informed consent. The experiment was performed in accordance with the Declaration of Helsinki and was approved by the Ethics Committee of Southwest University.

### Experimental design

The transient auditory stimuli were 1 ms monotone pulses, and the quasi-steady-state auditory stimuli were descending and ascending trains of 1 ms monotone pulses (101 pulses for each train, i.e., P1, P2, …, P101; Fig. 1). All auditory stimuli were presented at a comfortable listening level (~80 dB SPL) through binaural earphones. As displayed in Fig. 1, the inter-pulse intervals (IPIs) in the descending train were changed from 10 ms to 1000 ms (1000/100 ms between P1 and P2, 1000/99 ms between P2 and P3, 1000/98 ms between P3 and P4, …, 1000/1 ms between P100 and P101). In this type of train, the stimulus frequencies were 100, 99, 98, …, 1 Hz between the consecutive pulses. The IPIs in the ascending train were changed from 1000 ms to 10 ms (1000/1 ms between P1 and P2, 1000/2 ms between P2 and P3, 1000/3 ms between P3 and P4, …, 1000/100 ms between P100 and P101). In this type of train, the stimulus frequencies were 1, 2, 3, …, 100 Hz between the consecutive pulses.

The whole experiment was comprised of 10 blocks, each of which lasted approximately 5 minutes and contained 45 auditory stimuli (i.e., 15 transient stimuli, 15 descending trains of quasi-steady-state stimuli, and 15 ascending trains of quasi-steady-state stimuli). The order of all auditory stimuli in each block was randomized for each subject. In total, there were 150 auditory stimuli for each of the three types. The inter-stimulus interval (ISI) varied randomly from 4 to 6 s, and 2–3 minutes break was taken between the consecutive blocks.

### EEG recording

Subjects were seated comfortably in a chair in a sound-attenuated, temperature-controlled room. Subjects were instructed to avoid gross movements, and were asked to relax their muscles and focus their attention on the auditory stimuli. Electroencephalographic (EEG) data were recorded using 64 Ag-AgCl scalp channels placed according to the International 10–20 system (Brain Products GmbH, Munich, Germany; pass band: 0.01–100 Hz; sampling rate: 500 Hz). The left mastoid (A1) was used as the reference channel, and all channel impedances were kept lower than 10 kΩ. To monitor ocular movements and eye blinks, electro-oculographic (EOG) signals were simultaneously recorded using four surface electrodes, one pair placed over the higher and lower eyelid, the other pair placed 1 cm lateral to the outer canthus of the left and right eyes.

### EEG data analysis

#### EEG data preprocessing

EEG data were processed using EEGLAB^41^, an open source toolbox running in the MATLAB environment, and in-house MATLAB functions. Continuous EEG data were band-pass filtered between 1 and 100 Hz. For transient auditory stimuli, EEG epochs were extracted using a window analysis time of 800 ms (from −200 ms to 600 ms) and baseline corrected using the pre-stimulus interval (−200–0 ms). For both types of quasi-steady-state auditory stimuli, EEG epochs were extracted using a window analysis time of 7500 ms (from −1000 ms to 6500 ms) and baseline corrected using the pre-stimulus interval (−1000–0 ms). Trials contaminated by eye-blinks and movements were corrected using an Independent Component Analysis algorithm^41^. In all datasets, these independent components had a large EOG channel contribution and a frontal scalp distribution. After artifact rejection and baseline correction, EEG epochs were re-referenced to the bilateral mastoid electrodes (A1 and A2).

#### Time domain analysis

For each subject and each stimulus type (transient stimuli, descending train of quasi-steady-state stimuli, and ascending train of quasi-steady-state stimuli), artifact-removed EEG epochs were averaged, time-locked to the onset of auditory stimuli. Single-subject average waveforms were subsequently averaged to obtain the group-level waveforms. Group-level scalp topographies were computed by spline interpolation. Peak latencies and amplitudes of N1 and P2 evoked by transient auditory stimuli were measured from the average waveform (FCz-A1A2) for each subject. To assess the significant difference between nonmusicians and musicians, each of these measured parameters were compared using an independent sample t-test with a statistical significance level of 0.05. In addition, we performed the same independent sample t-test, but used each time point of the averaged ERP waveforms evoked by transient auditory stimuli, which yielded a time course of P values, representing the significant level of difference between nonmusicians and musicians, for each channel.

#### Time-frequency analysis

A time-frequency distribution (TFD) of the EEG epoch was obtained using a windowed Fourier transform (WFT) with a fixed 200-ms Hanning window. The WFT yielded, for each epoch, a complex time-frequency estimate *F*(*t, f*) at each point (*t, f*) of the time-frequency plane, extending from −200 to 600 ms for neural responses to transient stimuli and from −1000 to 6500 ms for neural responses to quasi-steady-state stimuli (in steps of 2 ms) in the time domain, and from 1 to 100 Hz (in steps of 1 Hz) in the frequency domain. The resulting spectrogram, *P*(*t, f*)=|*F*(*t, f*)|^2^, represents the signal power as a joint function of time and frequency at each time-frequency point. When the WFT was applied to across-trial averages of the response in the time domain, the obtained TFDs only comprise brain responses phase-locked to stimulus onsets (evoked TFDs). When the same WFT was applied to single-trial EEG responses, the obtained TFDs comprise brain responses both phase-locked and non-phase-locked to stimulus onsets (induced TFDs).

To distinguish between phase-locked and non-phase-locked EEG responses, we calculated the phase-locking value (PLV)^29^, for each subject, as follows:
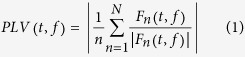
where *N* is the number of trials.

To test whether evoked TFDs, induced TFDs, and PLVs within the post-stimulus interval were significantly different from those within the pre-stimulus interval, we performed a bootstrapping test^41^,^42^,^43^. At each time-frequency point (*t, f*), we extracted a collection of numerical samples from the 28 subjects, and compared with a similar collection of numerical samples in the pre-stimulus interval (note that the pre-stimulus interval was −150 to −50 ms relative to the onset of transient stimuli and −800 to −200 ms relative to the onset of quasi-steady-state stimuli). The null hypothesis was that there was no difference between the means of the two numerical samples, i.e., no difference between the mean amplitude values within post- and pre-stimulus intervals. The pseudo-t statistic of two populations was calculated, and its probability distribution was estimated by permutation testing (5000 times). The distribution of the pseudo-t statistics from the baseline population was obtained, and the bootstrap P values for the null hypothesis were generated. This procedure identified the time-frequency regions where the magnitudes of TFDs were significantly different relative to the baseline interval^43^,^44^. To account for multiple comparisons, the significance level (expressed as P value) was corrected using an FDR procedure^45^.

Evoked TFDs, induced TFDs, and PLVs were baseline-corrected (reference interval: −150 to −50 ms relative to the onset of transient stimuli and −800 to −200 ms relative to the onset of quasi-steady-state stimuli) at each frequency *f* using subtraction approach^46^. The reference interval was chosen to avoid the adverse influence of spectral estimates biased by windowing post-stimulus activity and padding values^46^.

#### ROI based statistical analysis

For each of the baseline-corrected TFDs (evoked TFDs, induced TFDs, and PLVs), we performed a point-by-point independent sample t-test between nonmusicians and musicians to explore the time-frequency regions in which the baseline-corrected TFDs coded the significant difference between the two groups.

To account for the multiple comparison problem in the point-by-point statistical analysis of TFDs^47^, significant time-frequency pixels were grouped into a ROI based on their adjacency in the time-frequency plane (cluster-level statistical analysis). The definition of ROI for the subsequent quantitative analysis was based on the following three criteria: (1) TFD magnitudes within the ROI were significantly different than the magnitudes at the pre-stimulus interval (assessed using the above bootstrapping test); (2) TFD magnitudes within the ROI showed significant difference between nonmusicians and musicians (assessed using the point-by-point independent sample t-test); (3) only the ROI with larger than 400 significant time-frequency pixels were selected to control for false-positive observations^47^. Also, only the ROI with the largest number of significant time-frequency pixels in the low-frequency region (<30 Hz) and the ROI with the largest number of significant time-frequency pixels in the high-frequency region (≥30 Hz) were selected for the subsequent demonstrations^48^.

To verify the significant difference between nonmusicians and musicians within the selected ROIs, we performed the nonparametric permutation testing (5000 times) for each of the baseline-corrected TFDs^48^. In detail, the same independent sample t-test was performed at each time-frequency point of each ROI in each permutation, which yielded a ROI-level statistics (t values). Permutation distributions of the ROI-level t-statistics were obtained, and the two-tailed P value was obtained by locating the observed t value under the estimated permutation distribution. Once the significance was confirmed by permutation testing, the magnitudes of the baseline-corrected TFDs within each ROI were measured by computing the mean of all included time-frequency points for each subjects, and were compared between nonmusicians and musicians using an independent sample t-test. The group-level scalp topography of the magnitudes of the baseline-corrected TFDs within each ROI was computed by spline interpolation. It should be noted that the comparison of TFDs between ascending and descending trains of quasi-steady-state auditory stimuli was not performed due to the technical difficulty in temporal alignment of both responses.

## Additional Information

**How to cite this article**: Zhang, L. *et al.* Electrophysiological evidences demonstrating differences in brain functions between nonmusicians and musicians. *Sci. Rep.* **5**, 13796; doi: 10.1038/srep13796 (2015).

## Figures and Tables

**Table 1:** Demographic characteristics of the nonmusicians and musicians, as well as the music training histories of the musicians.

Nonmusicians		Musicians				
*Sex*	*Age*	*Sex*	*Age*	*Instruments*	*Start training age*	*Years of training*
M	20	F	19	Erhu	10	9
M	19	F	19	Piano	11	8
F	20	M	18	Piano	8	5
M	21	M	20	Saxophone	13	7
F	23	M	22	Piano, Violin	10	5
M	22	F	19	Piano, Erhu	16	3
F	22	M	22	Erhu	12	10
F	22	M	20	Piano	10	9
M	21	M	19	Piano, Trombone	4	15
F	21	M	20	Piano	18	2
M	21	M	21	Saxophone, Bassoon	8	13
F	21	M	18	Piano	8	10
M	20	F	24	Piano	12	12
M	21	F	25	Electronic Organ, Piano	6, 13	19, 12
